# Extreme temperature and mood disorders: A systematic review of literature

**DOI:** 10.1192/j.eurpsy.2025.611

**Published:** 2025-08-26

**Authors:** N. Manoj, M. K. Kennedy, M. Liu, A. T. Olagunju

**Affiliations:** 1 Michael G. DeGroote School of Medicine; 2 Department of Psychiatry and Behavioural Neurosciences, McMaster University; 3 Forensic Psychiatry Program, St. Joseph’s Healthcare Hamilton, Hamilton, Canada; 4 Department of Psychiatry and Behavioral Sciences, University of Oklahoma, Oklahoma City, United States; 5 Discipline of Psychiatry, The University of Adelaide, Adelaide, Australia

## Abstract

**Introduction:**

The prevalence of extreme temperature is increasing largely due to the progression of climate change globally (LaSorte *et al.* Climate Change 2021; 166 1-2). Existing research indicates extreme temperatures have an impact on mental health, including its effect on mood disorders (Rony & Alamgir. Health Sci Rep 2023; 6 12). While there is evidence to suggest that mood disorders can be influenced by various environmental, biological, and social factors (Zhang *et al.* Environmental International 2020; 143), no study has synthesized findings on the relationship between extreme temperature and mood disorders in existing literature.

**Objectives:**

The study aims to: investigate the linkage between extreme temperature and mood disorders in terms of symptom severity, hospital admissions and adverse events; describe factors moderating the relationship between extreme temperature and mood disorders; outline study-defined interventions and make policy recommendations.

**Methods:**

This review was conducted following the Preferred Reporting Items for Systematic Reviews and Meta-Analyses (PRISMA) guideline. Major databases (Medline/PubMed, PsychINFO, Scopus, Web of Science) were searched for eligible reports using a search strategy developed for the study. This was supplemented by snowball searching for references in relevant studies. Title and abstract screening and data extraction were completed by at least two independent investigators and conflicts were resolved by discussion amongst investigators or consulting the senior author. All included studies will be assessed with the National Institutes of Health Study Quality Assessment Tools.

**Results:**

As seen in Image 1, 468 articles were identified from searching databases. Following screening and full-text review, 22 articles were selected for data extraction. Preliminary findings showed that the included studies were conducted in North America, Europe, Asia, and Oceania-Australia among others. The included studies were of different designs, including case-crossover, cohort and cross-sectional studies. Findings across studies indicate that extreme temperatures have a complex and significant impact on mood disorders. High temperatures were associated with increased hospital admissions, with adolescents, women, and the elderly especially vulnerable. Individuals with bipolar disorder and depression showed increased sensitivity to heat exposure. While some studies found increased emergency department visits for mood disorders during periods of extreme heat, others revealed insignificant correlations. Moreover, short-term exposure to humidity was also linked to elevated risk for mood disorders.

**Image 1:**

**Figure:**
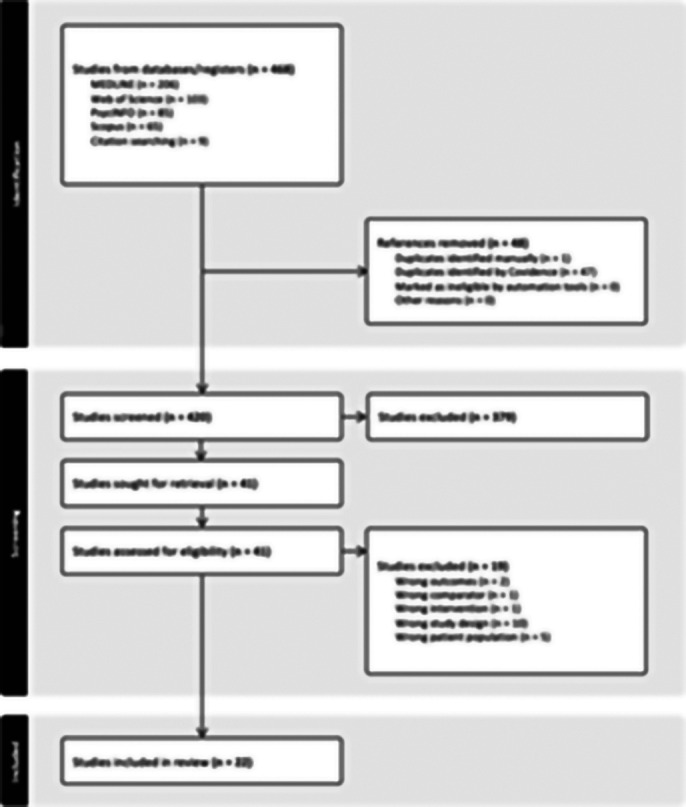

**Conclusions:**

This study underscores the impact of extreme temperatures on mood disorders and highlights the need for real-world solutions, like policy implementation, to reduce exposure to such conditions due to climate change.

**Disclosure of Interest:**

None Declared

